# Lactobacillus‐Based Microbiome Therapy for Acne Vulgaris: A GRADE Systematic Review and Meta‐Analysis of Randomized Controlled Trials

**DOI:** 10.1111/jocd.70792

**Published:** 2026-03-19

**Authors:** Zain Ul Abedin, Asim Shah, Safa Mazhar, Saim Mahmood Khan, Ahmad Bin Aamir, Sheeza Yousaf, Deeksha Fnu, Raghabendra Kumar Mahato, Asma Ansari

**Affiliations:** ^1^ King Edward Medical University Lahore Pakistan; ^2^ Khyber Medical College Peshawar Pakistan; ^3^ Jinnah Sindh Medical University Karachi Pakistan; ^4^ Karachi Medical and Dental College Karachi Pakistan; ^5^ Punjab Medical College Faisalabad Medical University Faisalabad Pakistan; ^6^ Post Graduate Institute of Medical Education and Research Chandigarh India; ^7^ Gandaki Medical College Teaching Hospital and Research Centre Pokhara Nepal; ^8^ Dow University of Health Sciences Karachi Sindh Pakistan

**Keywords:** acne, acne vulgaris, GRADE approach, inflammatory lesions, lactobacillus probiotics, meta‐analysis, microbiome, non‐inflammatory lesions, randomized controlled trials, systematic review

## Abstract

**Background:**

Acne vulgaris is one of the most prevalent disorders affecting 9%–10% of the global population, representing as papules, pustules, and comedones, with a pathogenesis involving increased sebum production, C. acnes colonization, and inflammation. Conventional treatments like retinoids and antibiotics often cause side effects, thus diverting attention toward probiotics as an alternative therapy. Lactobacillus probiotics, having their immunomodulatory, anti‐inflammatory, and antimicrobial properties, are useful in managing acne by reducing inflammation and oxidative stress with proved safety profile and the potential to reduce antibiotic reliance. This systematic review and meta‐analysis evaluate the efficacy of Lactobacillus‐based probiotics compared to placebo and benzoyl peroxide in reducing inflammatory lesions, non‐inflammatory lesions, and total acne lesion counts. The findings aim to clarify their therapeutic role and provide evidence on their effectiveness and safety.

**Objectives:**

This systematic review and meta‐analysis investigated the effectiveness of oral and topical Lactobacillus‐based probiotics or postbiotics, compared with placebo or benzoyl peroxide, in patients with acne vulgaris.

**Methods:**

A systematic review and meta‐analysis of randomized controlled trials (RCTs) was conducted, including studies evaluating oral or topical Lactobacillus‐based probiotic or postbiotic interventions in patients with acne vulgaris. Primary outcomes were changes in inflammatory lesion counts, while secondary outcomes included non‐inflammatory and total lesion counts, skin hydration, and sebum concentration. All analyses were performed using random‐effects models with 95% confidence intervals (CI), and heterogeneity was quantified using the I^2^ statistic.

**Results:**

A total of five RCTs involving 332 participants were included. The pooled mean difference for non‐inflammatory lesions was −1.39 (95% CI −5.10 to 2.32, *p* = 0.46), for inflammatory lesions was −0.08 (95% CI −1.28 to 1.11, *p* = 0.89), and for total lesion counts was −9.07 (95% CI −20.71 to 2.57, *p* = 0.13). These results concluded that there was no significant reduction in lesion counts with Lactobacillus‐based probiotics as compared to placebo or benzoyl peroxide. Heterogeneity was moderate to low across studies.

**Conclusion:**

This meta‐analysis indicates that Lactobacillus‐based probiotics do not provide significant clinical benefits in reducing inflammatory lesions, non‐inflammatory lesions, and total acne lesion counts in Acne vulgaris patients compared to placebo or benzoyl peroxide.

## 1 Introduction

Acne vulgaris is one of the most prevalent dermatological disorders, the eighth most common disease worldwide, affecting approximately 9%–10% of the global population [1]. Acne presents as comedones, papules, pustules, and nodules, and primarily affects adolescents but can persist into adulthood [2, 3]. Its multifactorial pathogenesis involves increased sebum production, follicular hyperkeratinization, *Cutibacterium acnes* colonization, and inflammatory immune responses [4]. Beyond physical manifestations, acne also impairs psychosocial health, often leading to anxiety, depression, and reduced quality of life [5].

First‐line therapies for acne include topical retinoids, benzoyl peroxide, and antibiotics, whereas systemic agents, such as isotretinoin and hormonal therapies, are reserved for moderate to severe disease [6]. However, these conventional treatments often cause cutaneous irritation, systemic side effects, and antimicrobial resistance [7, 8]. Hence, considerable research efforts are focused on adjunctive or alternative approaches that are safe, tolerable, and effective [9]. Among these therapies, probiotics targeting the skin–gut axis have shown promising results in management [10].

Probiotics are defined as live microorganisms that, when administered in adequate amounts, confer a health benefit to the host through their immunomodulatory, anti‐inflammatory, and antimicrobial properties [11]. *Lactobacillus* species help in the management of acne by inhibiting *C. acnes* growth, enhancing skin barrier function, and reducing oxidative stress [12]. Probiotics attract clinical attention given their safety profile and the potential to reduce antibiotic reliance [13].

Several randomized controlled trials (RCTs) have evaluated *Lactobacillus*‐containing probiotics in patients with acne, either as monotherapy or in combination with conventional agents. While some studies reported significant reductions in inflammatory and non‐inflammatory lesions when compared with placebo or benzoyl peroxide, others found modest or non‐significant effects [1, 14, 15]. Variability in probiotic strains, formulations, treatment durations, and outcome definitions contributes to heterogeneity across trials [16]. Previous reviews have comprehensively discussed probiotics as adjunctive acne therapy, but the inclusion of varied classes of probiotics limits the clarity of conclusions [17, 18]. To date, studies centered on *Lactobacillus*‐based probiotics remain limited, underscoring the need for focused evaluation using a standardized method like the GRADE framework.

We therefore conducted a systematic review and meta‐analysis of RCTs using the GRADE framework to evaluate the effectiveness of *Lactobacillus*‐based probiotics in acne vulgaris, compared with placebo or benzoyl peroxide. Primary outcomes were reductions in inflammatory lesions, non‐inflammatory lesions, and total acne lesion counts. This review aims to clarify the therapeutic role of *Lactobacillus* probiotics in acne management and provide an evidence‐based assessment of their efficacy and safety.

## 2 Methods

This review was conducted as per the Preferred Reporting Items for Systematic Reviews and Meta‐Analyses (PRISMA) 2020 guidelines [19], and the protocol was prospectively registered in the International Prospective Register of Systematic Reviews (PROSPERO) (CRD420251151917).

### 2.1 Literature Sources and Search Strategy

We systematically searched PubMed, Embase, Cochrane Central Register of Controlled Trials (CENTRAL), ClinicalTrials.gov, Web of Science, and Scopus from their inception to August 20, 2025. The search strategy combined controlled vocabulary and free‐text terms (“Acne Vulgaris”[Mesh] OR “Acneiform Eruptions”[Mesh] OR) AND (“Probiotics”[Mesh] OR “Lactobacillus”[Mesh] OR “Lactic Acid Bacteria” [Mesh] OR “Microbiota”[Mesh] OR “Gastrointestinal Microbiome”[Mesh] OR “Dietary Supplements”[Mesh] OR “Bacterial Products”[Mesh]). In addition, reference lists of relevant reviews and included studies were screened manually to identify further eligible trials. Searches were limited to English‐language publications to ensure accuracy due to the unavailability of translation resources.

### 2.2 Eligibility Criteria

Studies were eligible if they met the following inclusion criteria: Randomized controlled trials (parallel or crossover design) enrolling participants of any age and acne severity with a clinical diagnosis of acne vulgaris, conducted in outpatient or community settings. Interventions had to involve *Lactobacillus*‐based probiotics or postbiotics administered orally or topically, either as single‐strain products or as part of multi‐strain formulations in which *Lactobacillus* was explicitly reported. Eligible comparators were placebo, benzoyl peroxide, or standard dermatologic care without additional active anti‐acne agents. To be included, trials were required to report extractable data for inflammatory lesion counts, which constituted the primary outcome. Studies that reported total lesion counts and/or non‐inflammatory lesion counts were eligible for secondary analyses. Only human studies with a minimum treatment or follow‐up duration of three or more weeks were included.

Non‐randomized studies, including observational designs, case series, case reports, in vitro studies, and animal experiments, were excluded. Trials were ineligible if *Lactobacillus* was not part of the probiotic or postbiotics formulation or if the effect of *Lactobacillus* could not be isolated. Studies were also excluded if the comparator did not include a placebo or benzoyl peroxide, unless the probiotic effect could be isolated from co‐interventions. Trials without extractable outcome data were excluded if missing information could not be obtained after reasonable attempts to contact study authors. In cases of duplicate publications, the most comprehensive dataset was retained, and redundant reports were excluded.

### 2.3 Study Selection and Data Extraction

Two reviewers (Z.A and S.K) independently screened titles and abstracts, followed by a full‐text review of potentially eligible articles. Discrepancies were resolved by discussion or consultation with a third reviewer (S.Y). Data were extracted using a standardized form that included study characteristics: First author, year, country, design, setting, sample size, and treatment duration. Participant characteristics included age, sex, BMI, acne severity, baseline lesion counts, allergies, and smoker status. Intervention details like probiotic strain, formulation, dose, route, frequency, and duration were studied; comparators were placebo or benzoyl peroxide.

### 2.4 Risk of Bias Assessment

Included trials were assessed for their methodological quality individually by two reviewers (S.K and S.M) using the Cochrane Risk of Bias 2.0 (RoB 2) tool [20]. This tool evaluates five domains: The randomization process, deviations from intended interventions, missing outcome data, outcome measurement, and selective reporting. Each study was judged as having a “low risk of bias,” “some concerns,” or “high risk of bias.” Differences in assessments were resolved by discussion. The RoB 2 tool was applied per the Cochrane Handbook guidelines [21].

### 2.5 Outcomes

The primary outcome was the change in inflammatory acne lesion count from baseline to the end of treatment. Secondary outcomes included any change in total lesion count or non‐inflammatory lesion count, skin hydration, and sebum concentration. Where outcome data were reported as standard errors (SE) or 95% confidence intervals (CI), these were converted to standard deviations (SD) using validated formulas as described in the Cochrane Handbook for Systematic Reviews of Interventions [21].

### 2.6 Statistical Analysis

All analyses were performed using random‐effects models (DerSimonian–Laird method) to account for clinical and methodological heterogeneity. For continuous outcomes, pooled mean differences (MD) with 95% confidence intervals (CI) were calculated. Heterogeneity was quantified using the I^2^ statistic, with values of 25%, 50%, and 75% interpreted as low, moderate, and high heterogeneity, respectively. Sensitivity analyses were planned by excluding studies with a high risk of bias. Publication bias was assessed using funnel plots and Egger's regression test when at least ten studies were available for a given outcome.

### 2.7 Certainty of Evidence

The certainty of evidence was assessed using the Grading of Recommendations Assessment, Development and Evaluation (GRADE) framework [22]. This approach evaluates five domains: Risk of bias, inconsistency, indirectness, imprecision, and publication bias. Evidence was rated as high, moderate, low, or very low certainty. To enhance clarity and transparency, the results were organized into a GRADE Summary of Findings table.

### 2.8 Subgroup Analysis

We performed subgroup analyses stratified by comparator type in addition to the primary pooled analysis. Trials were categorized as either placebo‐controlled or benzoyl peroxide–controlled, allowing us to assess whether the choice of control influenced treatment effects and tolerability outcomes.

## 3 Results

### 3.1 Search Results

Database searches yielded 525 records in total (PubMed = 214, Cochrane = 193, Embase = 83, ClinicalTrials.gov = 35). 472 unique articles were left for screening after 53 duplicates were eliminated. 427 articles were eliminated after screening for titles and abstracts because they did not fit the requirements for inclusion. After evaluating the full texts of the remaining 45 studies for eligibility, 40 were disqualified (16 were not randomized controlled trials, 8 looked into populations that did not have acne, 7 combined probiotics with pharmaceutical medications, and 9 did not report outcomes related to acne). Lastly, this systematic review and meta‐analysis included five randomized controlled trials.

At the end of the screening process, five randomized controlled trials met the inclusion criteria and were included in this systematic review and meta‐analysis (Eguren 2023, Shi 2024, Kim 2021, Sathikulpakdee 2021, Majeed 2020) [1, 14, 23, 24, 25]. This selection process is shown in the PRISMA flow diagram (Figure S1, supplementary files).

### 3.2 Study and Patient Characteristics

All included studies were randomized controlled trials. The five trials contributed a total of 332 participants (intervention *n* = 169; comparator *n* = 163). Study locations were Spain (Eguren 2023), China (Shi 2024), Korea (Kim 2021), Thailand (Sathikulpakdee 2021), and India (Majeed 2020) [1, 14, 23, 24, 25]. Interventions were probiotics containing Lactobacillus species (including CJLP55 and Lactosporin formulations); comparators were placebo in three trials (Eguren, Shi, Kim) and topical benzoyl peroxide (2.5%) in two trials (Sathikulpakdee, Majeed). Study sample sizes per arm ranged from 14 to 52 participants. Baseline patient characteristics are summarized in Table S2 of the supplementary files; mean ages across studies ranged from about 20 to 29 years. Where reported, baseline total lesion counts and inflammatory lesion counts were broadly similar between intervention and comparator groups (Table S1, supplementary files).

### 3.3 Meta‐Analysis Outcomes

#### 3.3.1 Non‐Inflammatory Acne Lesions

Four randomized trials (*n* = 317; 160 probiotic, 157 control) reported change in non‐inflammatory lesion counts and were pooled using a random‐effects inverse‐variance model. The pooled mean difference was −1.39 lesions (95% CI −5.10 to 2.32), indicating a non‐statistically significant trend toward fewer non‐inflammatory lesions with Lactobacillus‐based probiotics. Heterogeneity was low–moderate (Tau^2^ = 3.19; Chi^2^ = 3.81, df = 3, *p* = 0.28; I^2^ = 21%), and the test for overall effect was not significant (Z = 0.73, *p* = 0.46). In summary, current trials do not provide clear evidence that Lactobacillus‐based probiotics reduce non‐inflammatory acne lesion counts compared with controls. Figure 1A Forest plot for non‐inflammatory acne lesions.

**FIGURE 1A:**
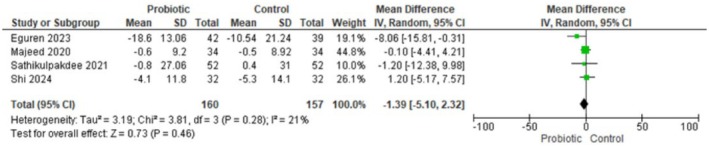
Forest plot showing no significant difference in non‐inflammatory acne lesions between probiotics and control.

**FIGURE 1B:**
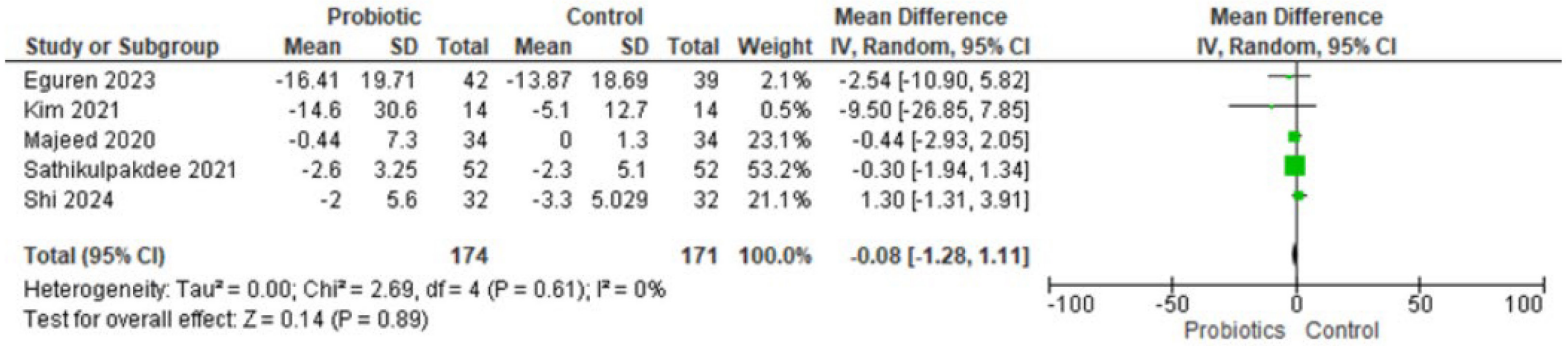
Forest plot for inflammatory acne lesions. Figure 1B Forest plot showing no reduction in inflammatory lesions with probiotic treatment vs. control.

**FIGURE 1C:**
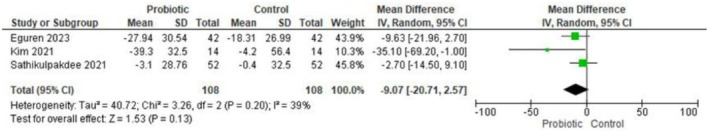
Forest plot for total lesion counts. Figure 1C Forest plot showing a nonsignificant trend favoring probiotics for total lesion counts.

**FIGURE 1D:**
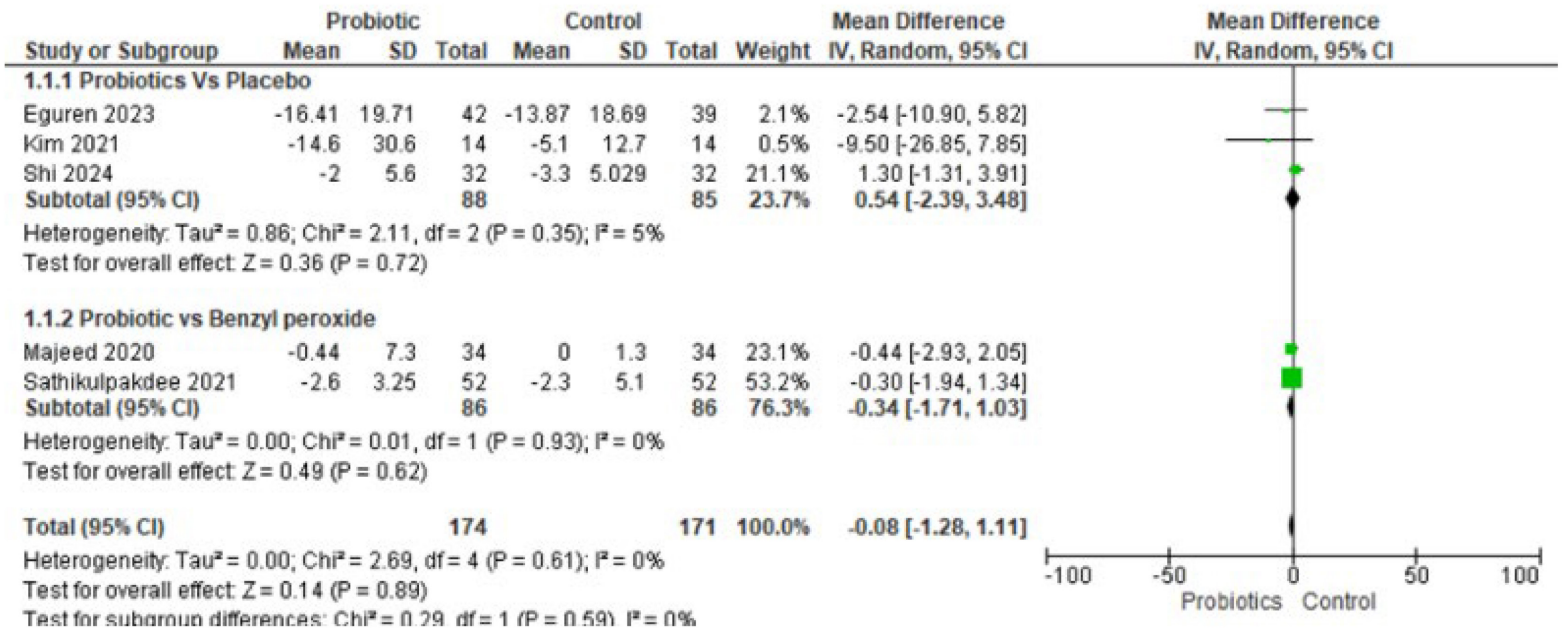
Subgroup analyses of Inflammatory Acne. Figure 1D Subgroup analysis showing no difference in inflammatory lesions by comparator type.

**FIGURE 1E:**
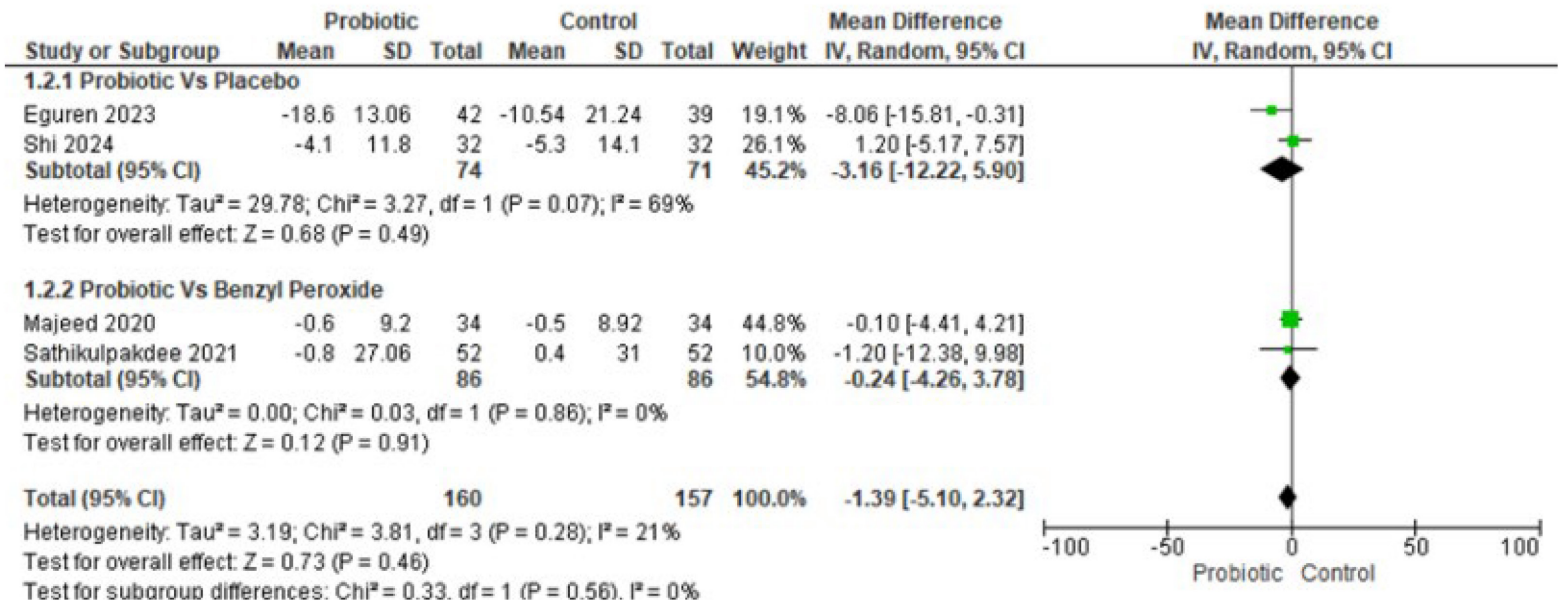
Subgroup analyses of non‐Inflammatory Acne. Figure 1E Subgroup analysis showing inconsistent, nonsignificant effects for non‐inflammatory lesions.

#### 3.3.2 Inflammatory Acne Lesions

Five randomized trials (*n* = 345; 174 probiotic, 171 control) contributed data on change in inflammatory acne lesion counts. Using a random‐effects inverse‐variance model, the pooled mean difference was −0.08 lesions (95% CI −1.28 to 1.11), indicating no clear difference between Lactobacillus‐based probiotics and control. Heterogeneity was negligible (Tau^2^ = 0.00, Chi^2^ = 2.69, df = 4, *p* = 0.61; I^2^ = 0%), and the test for overall effect was not significant (Z = 0.14, *p* = 0.89). In short, there is no evidence from these trials that Lactobacillus‐based probiotics reduce inflammatory lesion counts compared with controls.

#### 3.3.3 Total Lesion Counts

Three trials reported total lesion counts (Eguren 2023, Kim 2021, Sathikulpakdee 2021) [1, 14, 23]. The pooled MD favored probiotics numerically but did not reach statistical significance: MD = −9.07 (95% CI −20.71 to 2.57), *p* = 0.13. Heterogeneity was low to moderate (I^2^ = 39%, *p* = 0.20). At the study level, only Kim 2021 [14] showed a statistically significant benefit favoring probiotics (MD −35.10, 95% CI −69.20 to −1.00).

#### 3.3.4 Subgroup Analyses of Inflammatory Acne

For inflammatory lesions, subgroup analyses stratified by comparator (placebo vs. benzoyl peroxide) did not reveal a clinically or statistically meaningful difference in effect estimates. When probiotics were compared with a placebo, the pooled mean difference was 0.54 (95% CI −2.39 to 3.48, *p* = 0.72; I^2^ = 5%), and when compared with benzoyl peroxide, the pooled mean difference was −0.34 (95% CI −1.71 to 1.03, *p* = 0.62; I^2^ = 0%). Both subgroup point estimates are small, and their confidence intervals overlap the null; heterogeneity within each subgroup was negligible. Taken together (all comparators), the overall pooled MD for inflammatory lesions remained essentially null (MD −0.08, 95% CI −1.28 to 1.11, *p* = 0.89; I^2^ = 0%), indicating no consistent evidence that Lactobacillus‐based probiotics reduce inflammatory lesion counts compared with either placebo or topical benzoyl peroxide.

#### 3.3.5 Subgroup Analyses of Non‐Inflammatory Acne

The pattern for non‐inflammatory lesions was more heterogeneous and merits cautious interpretation. In the placebo subgroup, the pooled MD was −3.16 (95% CI −12.22 to 5.90, *p* = 0.49) with substantial heterogeneity (I^2^ = 69%), whereas in the benzoyl peroxide subgroup, the pooled MD was −0.24 (95% CI −4.26 to 3.78, *p* = 0.91) with no observed heterogeneity (I^2^ = 0%). The larger point estimate favoring probiotics in the placebo subgroup suggests a possible benefit, but the wide confidence interval, lack of statistical significance, and especially the high between‐study inconsistency indicate that this finding is imprecise and likely influenced by study differences. By contrast, the benzoyl peroxide subgroup shows a near‐null effect with consistent results across the two contributing trials, suggesting that probiotics offer no clear advantage over benzoyl peroxide for non‐inflammatory lesions in the limited data available.

Overall, these subgroup results do not provide robust evidence of effect modification by comparator type.

### 3.4 Risk of Bias Assessment

Accessed via the Cochrane Risk of Bias 2.0 tool, the risk of bias was generally low across the five included trials. Two studies raised some concerns: Kim et al. (2021) was rated “some concerns” for deviations from the intended interventions (D2), and Majeed et al. (2020) was rated “some concerns” for selection of the reported result (D5); these domain‐level judgments led to an overall rating of “some concerns” for those two trials. The remaining three studies (Eguren et al., Sathikulpakdee et al., and Shi Z. et al.) were judged at low risk overall. No study was judged at high risk of bias in any domain. Taken together, the body of evidence is of generally low risk of bias. The complete details are present in Supplementary Figure S2 (A, B).

### 3.5 Grade Assessment

Overall, the GRADE assessments indicated high certainty for inflammatory acne when all RCTs were considered, and this high certainty was maintained in the subgroup comparisons of probiotics vs. placebo and probiotics vs. benzoyl peroxide. For non‐inflammatory acne, the overall rating across RCTs was high, although the probiotics vs. placebo subgroup was rated low and the probiotics vs. benzoyl peroxide subgroup moderate. Evidence for total lesion counts was rated moderate. It is thoroughly reported in Table S3 of the supplementary files.

## 4 Discussion

Our meta‐analysis suggests that lactobacillus‐based probiotics do not significantly reduce acne lesion counts, including the total number of lesions (MD = −9.07, 95% CI: −20.71 to 2.57, *p* = 0.13), inflammatory lesions (MD = −0.08, 95% CI: −1.28 to 1.11, *p* = 0.89), or non‐inflammatory lesions (MD = −1.39, 95% CI: −5.10 to 2.32, *p* = 0.46).

Our findings differ from those reported by Lin et al. [26], who concluded that probiotics significantly decreased all three lesion categories after a 12‐week intervention. Several factors may account for these divergent results. First, differences in probiotic strain selection may be influential. Our analysis focused exclusively on lactobacillus‐based probiotics, whereas Lin et al. [26] included a broader spectrum of probiotic strains. The therapeutic effects of probiotics are increasingly recognized as strain‐specific, with distinct strains exhibiting variable immunological and metabolic activities. For example, certain *Lactobacillus* strains have demonstrated efficacy in preventing antibiotic‐associated diarrhea, while others have not [27]. Similarly, the extent and nature of cytokine modulation appear to vary between strains [28]. Without careful strain‐level differentiation, clinical outcomes are likely to remain inconsistent. Second, variation in the route of administration may also contribute. While Lin et al. [26] restricted their analysis to oral supplementation, we included both oral and topical probiotic interventions. Differences in systemic absorption, bioavailability, and localized skin activity could introduce heterogeneity and influence overall treatment effects. Third, the duration of probiotic administration appears to be a critical determinant of efficacy. Lin et al. [26] observed no meaningful reductions in lesion counts at four weeks, but significant improvements at 12 weeks. In contrast, two of the five studies included in our analysis employed intervention periods of less than 4 weeks [23, 24]. This shorter duration may have limited the opportunity to observe clinically meaningful changes, particularly given the chronic and fluctuating nature of acne.

A possible explanation for the lack of significant findings in our study is that probiotics primarily target inflammation, which represents only one of many pathogenic factors in acne. Acne is a multifactorial condition influenced by hormonal imbalance, excessive sebum production, *Cutibacterium acnes* overgrowth, follicular hyperkeratinization, environmental exposures, lifestyle factors, and genetic predisposition [29]. Probiotics may reduce systemic and gut‐related inflammation by suppressing proinflammatory cytokines such as IFN‐γ, TNF‐α, IL‐4, and IL‐13, lowering circulating hs‐CRP, and enhancing anti‐inflammatory cytokines such as IL‐10 [30]. However, their inability to modulate other key drivers of acne pathogenesis likely limits their overall therapeutic impact. Probiotics may also act indirectly by modifying the gut microbiome; however, the gut–skin axis is highly individualized (30), which could explain why some individuals respond more favorably to specific probiotic strains than others.

## 5 Clinical Implications of the Results

From a clinical perspective, our findings suggest that lactobacillus‐based probiotics cannot yet be recommended as a reliable therapeutic option for acne management. Until larger, well‐designed randomized controlled trials with standardized strains, consistent routes of delivery, and sufficient intervention durations are available, the role of probiotics in acne treatment should be regarded as investigational rather than established.

## 6 Limitations

This study also has several limitations that should be acknowledged. The number of included trials was relatively small, and sample sizes within individual studies were limited, reducing the overall statistical power. Considerable heterogeneity was present across studies with respect to dosage, route of administration, and duration of intervention, which may have influenced the pooled results. Publication bias cannot be excluded, as studies with null or negative findings are less likely to be published. Finally, most studies did not stratify outcomes by important confounding factors such as diet, lifestyle, or hormonal status, which may affect both gut microbiota composition and acne severity. These limitations highlight the need for more robust, standardized clinical trials before firm conclusions can be drawn. Future studies should explore personalized probiotic interventions based on individual microbiome profiles, strain selection, and targeted anti‐inflammatory mechanisms to better elucidate their potential role in acne management.

## 7 Conclusion

Lactobacillus‐based probiotics do not significantly decrease total number of lesions (MD = −9.07, 95% CI = −20.71 to 2.57, *p* = 0.13), number of inflammatory acne lesions (MD = −0.08, 95% CI = −1.28 to 1.11, *p* = 0.89), and number of non‐inflammatory acne lesions (MD = −1.39, 95% CI = −5.10 to 2.32, *p* = 0.46). These results suggest that there is no clear evidence of efficacy for probiotics in reducing acne lesions.

## Author Contributions

Z.U.A. Conceptualization‐Lead, Data curation‐Lead, Formal analysis‐Lead, Funding acquisition‐Lead, Investigation‐Lead, Methodology‐Lead, Project administration‐Lead, Resources‐Lead, Software‐Lead, Supervision‐Lead, Validation‐Lead, Visualization‐Lead, Writing – original draft‐Lead, Writing – review and editing‐Lead. A.S. Conceptualization‐Lead, Data curation‐Lead, Formal analysis‐Lead, Funding acquisition‐Lead, Investigation‐Lead, Methodology‐Lead, Project administration‐Lead, Resources‐Lead, Software‐Lead, Supervision‐Lead, Validation‐Lead, Visualization‐Lead, Writing – original draft‐Lead, Writing – review and editing‐Lead. SM: Conceptualization‐Equal, Data curation‐Equal, Formal analysis‐Equal, Funding acquisition‐Equal, Investigation‐Equal, Methodology‐Equal, Resources‐Equal, Software‐Equal, Supervision‐Equal, Visualization‐Equal, Writing – original draft‐Equal, Writing – review and editing‐Equal. SMK: Conceptualization‐Equal, Data curation‐Equal, Formal analysis‐Equal, Funding acquisition‐Equal, Investigation‐Equal, Methodology‐Equal, Resources‐Equal, Software‐Equal, Writing – original draft‐Equal. ABA: Conceptualization‐Equal, Data curation‐Equal, Formal analysis‐Equal, Funding acquisition‐Equal, Investigation‐Equal, Methodology‐Equal, Project administration‐Equal, Writing – original draft‐Equal, Writing – review and editing‐Equal. SY: Conceptualization‐Equal, Data curation‐Equal, Formal analysis‐Equal, Funding acquisition‐Equal, Investigation‐Equal, Methodology‐Equal, Project administration‐Equal, Writing – original draft‐Equal. FD: Conceptualization‐Equal, Data curation‐Equal, Formal analysis‐Equal, Funding acquisition‐Equal, Investigation‐Equal, Methodology‐Equal, Writing – original draft‐Equal. RKM: Conceptualization‐Equal, Data curation‐Equal, Formal analysis‐Equal, Funding acquisition‐Equal, Software‐Equal, Writing – original draft‐Equal. AA: Conceptualization‐Equal, Formal analysis‐Equal, Funding acquisition‐Equal, Software‐Equal, Writing – original draft‐Equal.

## Funding

The authors have nothing to report.

## Ethics Statement

The authors have nothing to report.

## Consent

The authors have nothing to report.

## Conflicts of Interest

The authors declare no conflicts of interest.

## Supporting information


**Figure S1:** PRISMA Flow Diagram.
**Figure S2:** Risk And Bias Assessment.
S2(A) Traffic light.
**Table S1:** Characteristics of included Randomized Controlled Trials.
**Table S2:** Baseline Characteristics.
**Table S3:** GRADE Assessment.

## Data Availability

The data that support the findings of this study are available from the corresponding author upon reasonable request.
