# Supplementary material for: Lactobacillus‐Based Microbiome Therapy for Acne Vulgaris: A GRADE Systematic Review and Meta‐Analysis of Randomized Controlled Trials
Source: J Cosmet Dermatol. 2026 Mar 19;25(3):e70792. doi: 10.1111/jocd.70792 (PMC13000680; doi:10.1111/jocd.70792)
Supplement: Supplementary file 1 — Figure S1: PRISMA Flow Diagram. Figure S2: Risk And Bias Assessment. S2(A) Traffic light. Table S1: Characteristics of included Randomized Controlled Trials. Table S2: Baseline Characteristics. Table S3: GRADE Assessment. [file JOCD-25-e70792-s001.docx]

**Figure S1: Prisma Flow Diagram**

**
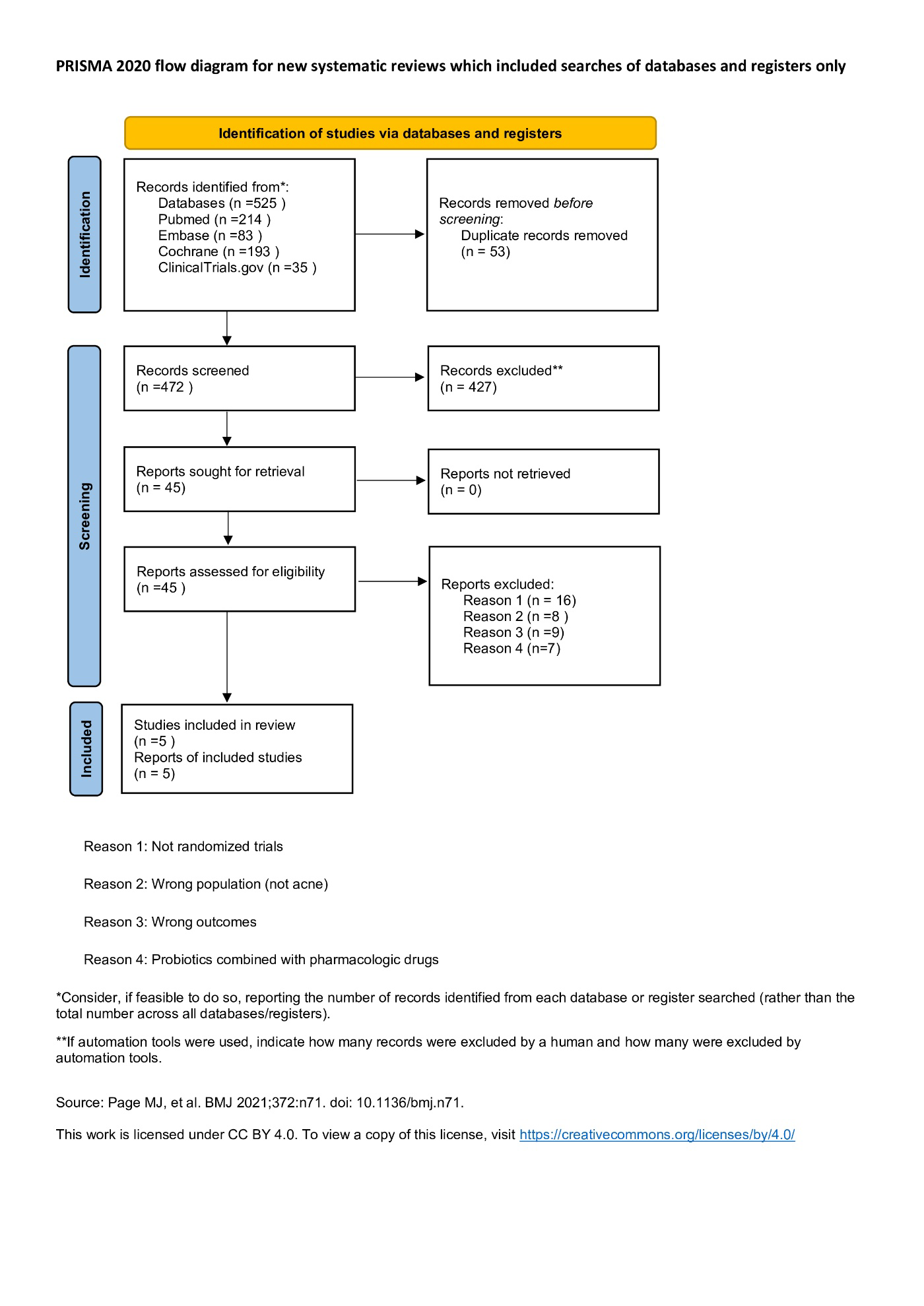
**

**Figure S2. Risk And Bias Assessment
S2(A) Traffic light**

**
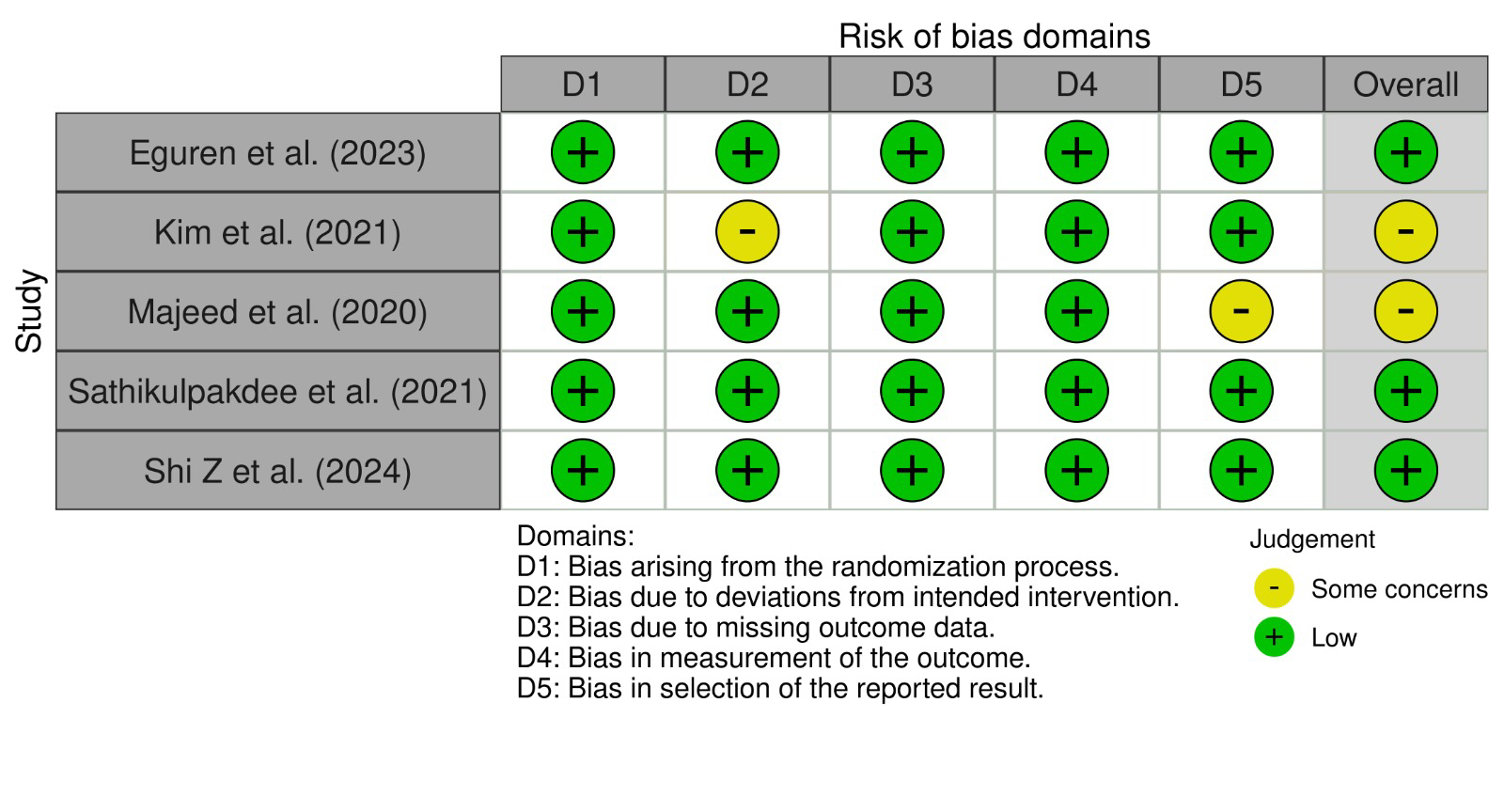
**

**S2(B)Summary Plot**

**
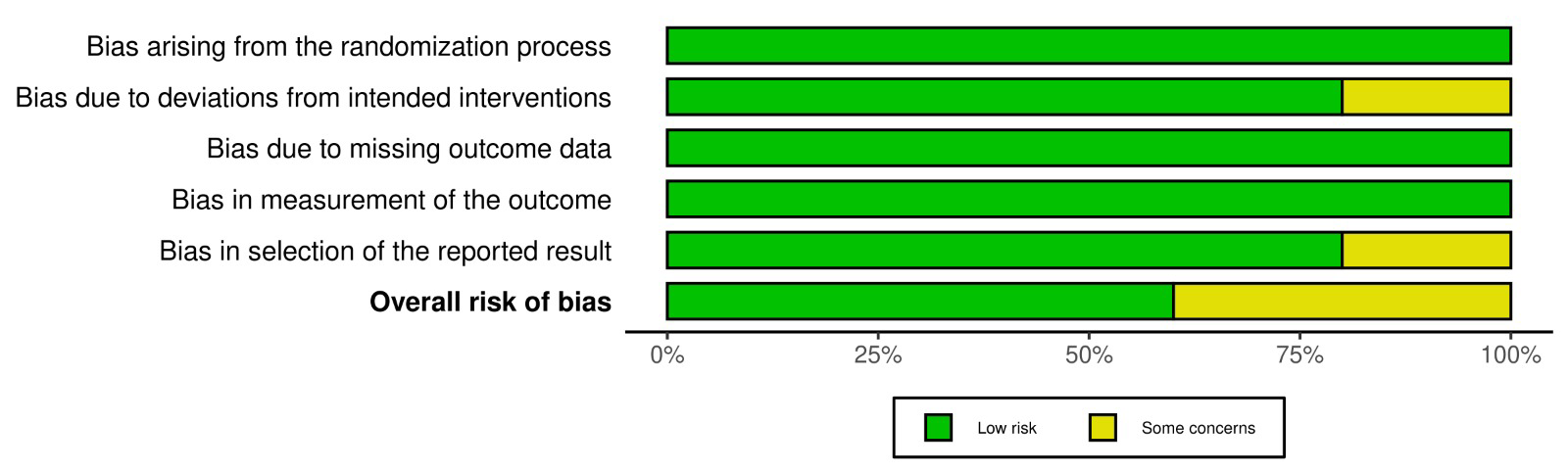
**

**Table S1: Characteristics of included Randomized Controlled Trials**

| **Study ID** | **COUNTRY** | **STUDY**  **TYPE & DURATION** | **ACNE SEVERITY** | **INTERVENTION** | **CONTROL** | **SAMPLE SIZE** | | **MALE(%)** | |
| --- | --- | --- | --- | --- | --- | --- | --- | --- | --- |
|  |  |  |  |  |  | **INTERVENTION** | **COMPARATOR** | **INTERVENTION** | **COMPARATOR** |
| EGUREN 2023 | SPAIN | RCT  (12 weeks) | MILD-SEVERE AGSS | PROBIOTIC | PLACEBO | 42 | 39 | 23.81 | 38.46 |
| SHI 2024 | CHINA | RCT  (8 weeks) | ALMOST CLEAR-MODERATE IGA | PROBIOTIC | PLACEBO | 29 | 26 | 0(ALL FEMALES) | 0 |
| KIM 2021 | KOREA | RCT  (12 weeks) | MILD-MODERATE IGA | CJLP55 | PLACEBO | 14 | 14 | 50 | 35.71 |
| SATHIKULPAKDEE 2021 | THAILAND | RCT  (4 weeks) | NOT GIVEN | PROBIOTIC | BENZYL PEROXIDE(2.5%) | 52 | 52 | 23.1 | 19.2 |
| MAJEEED 2020 | INDIA | RCT  (3 weeks) | MILD-MODERATE | POSTBIOTIC | BENZYL PEROXIDE(2.5%) | 32 | 32 | 56 | 47 |

**AGSS=Acne global severity scale**

**IGA=Investigators global assessment parameters**

**Table S2: Baseline Characteristics**

| **Study ID** | **Age,Years MEAN SD** | | **SMOKER %** | | **TOTAL LESION COUNT MEAN SD** | | **ALLERGY %** | | **INFLAMMATORY ACNE COUNT MEAN SD** |  | **SEBUM MEAN SD** | |
| --- | --- | --- | --- | --- | --- | --- | --- | --- | --- | --- | --- | --- |
|  | **intervention** | **comparater** | **INTERVENTION** | **COMPARATEO** | **INTERVENTION** | **COMPARATEO** | **INTERVENTION** | **COMPARATOR** | **INTERVENTION** | **COMPARATOR** | **INTERVENTION** | **COMPARATOR** |
| EGUREN 2023 | 20.13(5.04) | 18.03(5.28) | 16.67 | 5.13 | 90.57(54.04) | 90.72(52.32) | 2.38 | 5.13 | 21.36(14.52) | 28.54(23.18) | NR | NR |
| SHI 2024 | 29.1(1.9) | 29.1(1.9) | NR | NR | NR | NR | NR | NR | 8.8(1,0) | 9.8(1.0) | 103.4(5.4) | 95.9(4.8) |
| KIM 2021 | 24.29(0.73) | 23.86(0.80) | NR | NR | 72.93(10.11) | 98.14(15.75) | NR | NR | 17.79(3.19) | 19.64(3.81) | NR | NR |
| SATHIKULPAKDEE 2021 | 22.2(4.1) | 22.0(3.7) | NR | NR | 47.2(29.2) | 42.3(32.3) | NR | NR | 7.4(3.3) | 6.6(5.9) | NR | NR |
| MAJEEED 2020 | 23.7(2.1) | 24.9(2.1) | NR | NR | 41.94(24.71) | 38.12(21.76) | NR | NR | NR | NR | 106.74(9.78) | 108.13(9.32) |

**Table S3: Grade Assessment**

| **Outcomes compared to placebo for Acne**  **Bibliography: . Probiotics for Acne. Cochrane Database of Systematic Reviews [Year], Issue [Issue].** | | | | | | | | | | | |
| --- | --- | --- | --- | --- | --- | --- | --- | --- | --- | --- | --- |
| **Certainty assessment** | | | | | | | **Summary of findings** | | | | |
| **Participants (studies) Follow-up** | **Risk of bias** | **Inconsistency** | **Indirectness** | **Imprecision** | **Publication bias** | **Overall certainty of evidence** | **Study event rates (%)** | | **Relative effect (95% CI)** | **Anticipated absolute effects** | |
|  |  |  |  |  |  |  | **With placebo** | **With Outcomes** |  | **Risk with placebo** | **Risk difference with Outcomes** |
| **Inflammatory Acne** | | | | | | | | | | | |
| 345 (5 RCTs) | not serious | not serious | not serious | not serious | none | ⨁⨁⨁⨁ High | 171 | 174 | - | 171 | MD **0.08 lower** (1.28 lower to 1.11 higher) |
| **Inflammatory Acne - Probiotics Vs Placebo** | | | | | | | | | | | |
| 173 (3 RCTs) | not serious | not serious | not serious | not serious | none | ⨁⨁⨁⨁ High | 85 | 88 | - | 85 | MD **0.54 higher** (2.39 lower to 3.48 higher) |
| **Inflammatory Acne - Probiotic vs Benzyl peroxide** | | | | | | | | | | | |
| 172 (2 RCTs) | not serious | not serious | not serious | not serious | none | ⨁⨁⨁⨁ High | 86 | 86 | - | 86 | MD **0.34 lower** (1.71 lower to 1.03 higher) |
| **Non-Inflammatory Acne** | | | | | | | | | | | |
| 317 (4 RCTs) | not serious | not serious | not serious | not serious | none | ⨁⨁⨁⨁ High | 157 | 160 | - | 157 | MD **1.39 lower** (5.1 lower to 2.32 higher) |
| **Non-Inflammatory Acne - Probiotic Vs Placebo** | | | | | | | | | | | |
| 145 (2 RCTs) | not serious | serious^a^ | not serious | serious^b^ | none | ⨁⨁◯◯ Low^a,b^ | 71 | 74 | - | 71 | MD **3.16 lower** (12.22 lower to 5.9 higher) |
| **Non-Inflammatory Acne - Probiotic Vs Benzyl Peroxide** | | | | | | | | | | | |
| 172 (2 RCTs) | not serious | not serious | not serious | serious^b^ | none | ⨁⨁⨁◯ Moderate^b^ | 86 | 86 | - | 86 | MD **0.24 lower** (4.26 lower to 3.78 higher) |
| **Total Lesions** | | | | | | | | | | | |
| 216 (3 RCTs) | not serious | not serious | not serious | serious^b^ | none | ⨁⨁⨁◯ Moderate^b^ | 108 | 108 | - | 108 | MD **9.07 lower** (20.71 lower to 2.57 higher) |

**CI:** confidence interval; **MD:** mean difference

#### Explanations

a. High Heterogeneity = 69%

b. Wide CI suggests variability in the true effect.
